# Disruption of CCL20-CCR6 interaction inhibits metastasis of advanced cutaneous T-cell lymphoma

**DOI:** 10.18632/oncotarget.6916

**Published:** 2016-01-14

**Authors:** Sho Ikeda, Akihiro Kitadate, Mitsugu Ito, Fumito Abe, Miho Nara, Atsushi Watanabe, Naoto Takahashi, Tomomitsu Miyagaki, Makoto Sugaya, Hiroyuki Tagawa

**Affiliations:** ^1^ Department of Hematology, Nephrology, and Rheumatology, Akita University Graduate School of Medicine, Akita, Japan; ^2^ Department of Dermatology, University of Tokyo, Tokyo, Japan

**Keywords:** CCL20, CCR6, CTCL, STAT3, IL-22

## Abstract

We recently demonstrated that upregulation of a chemokine receptor CCR6 and its ligand CCL20 led to metastasis of advanced cutaneous T-cell lymphoma (CTCL) cells, suggesting the involvement of CCL20-CCR6 interaction in initiating CTCL cell metastasis. In this study, we determined whether this interaction is functional in metastatic CTCL cells. We first demonstrated increased STAT3 expression during the progression of primary CTCL. STAT3 was spontaneously activated and mediated the transcription of *CCL20* in CTCL cell lines. Next, to determine whether the transient knockdown of STAT3, CCL20, or CCR6 or treatment with neutralizing antibody against CCL20 (neutralizing CCL20 antibody) could reduce the migration ability of CTCL cells, we conducted an *in vitro* migration assay. All treatments reduced the nutrition-dependent migration activity of CTCL cells. Notably, treatment with neutralizing CCL20 antibody reduced the migration ability of the cells without decreasing the expression of CCL20 and CCR6. This demonstrated that the CCL20-CCR6 interaction is actually functional in metastatic CTCL cells. Finally, to examine the *in vivo* effect of neutralizing CCL20 antibody, we used NOD/Shi-scid IL-2γnul mice inoculated with CTCL cells. These mice were expected to die due to metastasis of CTCL cells into multiple organs. However, administration of neutralizing CCL20 antibody significantly prolonged the survival of the xenografted mice. These findings suggested that automatic activation of the STAT3/CCL20/CCR6 cascade was involved in CTCL lymphomagenesis and that disruption of CCL20-CCR6 interaction could be a key therapeutic strategy against advanced CTCL.

## INTRODUCTION

Chemokines are a family of low molecular weight (8-10 kDa) pro-inflammatory cytokines, which bind to chemokine receptors and sustain the migration of various leukocytes such as neutrophils, lymphocytes, monocytes, and dendritic cells. These molecules can be secreted by tumors, the adjacent stroma, and inflammatory cells, and play important roles in the occurrence and development of cancers [1]. They induce diverse cellular processes in tumors, such as proliferation, apoptosis and metastasis, by binding to the chemokine receptors [2]. Among these, the C-C chemokine CCL20, also known as macrophage infiltrating factor protein-3α (MIP-3α) or liver activation regulated chemokine (LARC), is the only known ligand for the receptor CCR6 [3, 4]. It has been reported that the expression levels of CCL20 and CCR6 are increased in many cancers such as gliomas, and colorectal and pancreatic cancers, and the increased expression of these molecules is closely associated with poor prognosis in cancer patients [5-8]. Experimental data have shown that CCL20-CCR6 interaction increases the colony-forming capacity of non-small cell lung cancer cells through upregulation of IL-17, and enhances hepatocellular carcinoma cell growth *via* activation of the ERK1/2 pathway [9, 10]. In thyroid cancers, CCL20-CCR6 interaction induces the activation of NF-κB, leading to migration activation [11]. CCL20-CCR6 interaction also stimulates the epithelial-mesenchymal transition (EMT) and metastasis of colorectal cancer *via* the PI3K/AKT-ERK1/2 signaling axis [7] and AKT signaling [12, 13] and promotes proliferation and invasion.

Cutaneous T-cell lymphoma (CTCL) is mainly comprised of mycosis fungoides (MF) and Sezary syndrome (SzS) [14-17]. MF is the most common form of CTCL and a good model for understanding the multistep process of cancer development and progression, owing to the clearly defined “early” and “advanced” stages [18]. Patients with early-stage MF (stage IA-IIA) have good prognosis with patch or plaque; however, advanced stages (stage IIB-IV) are associated with progression into erythroderma and multiple tumors, which are characterized by an aggressive clinical course with shortened survival and multiple metastases [15-19]. Between the early and advanced stages of MF, additional genetic or epigenetic alterations may occur, likely contributing to MF progression and aggressive clinical behavior. The expression of interleukin-22 (IL-22), chemokine receptor CCR6, and its ligand CCL20 is upregulated in advanced CTCL [20]. We have also shown that a non-coding RNA, microRNA-150 (miR-150), is silenced in advanced CTCL, and that the miR-150 downregulates CCR6 directly and CCL20 indirectly [21]. Based on these data, we hypothesized that continuous CCR6 and CCL20 upregulation might lead to continuous CCL20-CCR6 interaction in CTCL cells and in turn, lead to metastasis to distal organs in a nutrition-dependent manner. We further found that IL22RA1, one of the IL-22 receptor subunits, was aberrantly overexpressed in CTCL, and that its knockdown decreased CCL20 production [21]. This result suggested that the IL-22 produced by the CTCL cells might activate the IL-22 receptor in these cells, leading to the activation of downstream targets and subsequently increasing the transcription of *CCL20*. However, we could not determine the downstream cascade of IL-22 that mediates *CCL20* transcription activation. Therefore, in this study, we aimed to determine the molecule that mediates CCL20 activation and to elucidate whether CCL20-CCR6 interaction might be actually functional in metastatic CTCL.

## RESULTS AND DISCUSSION

### Upregulation of p-STAT3 during CTCL progression

**Figure 1:**
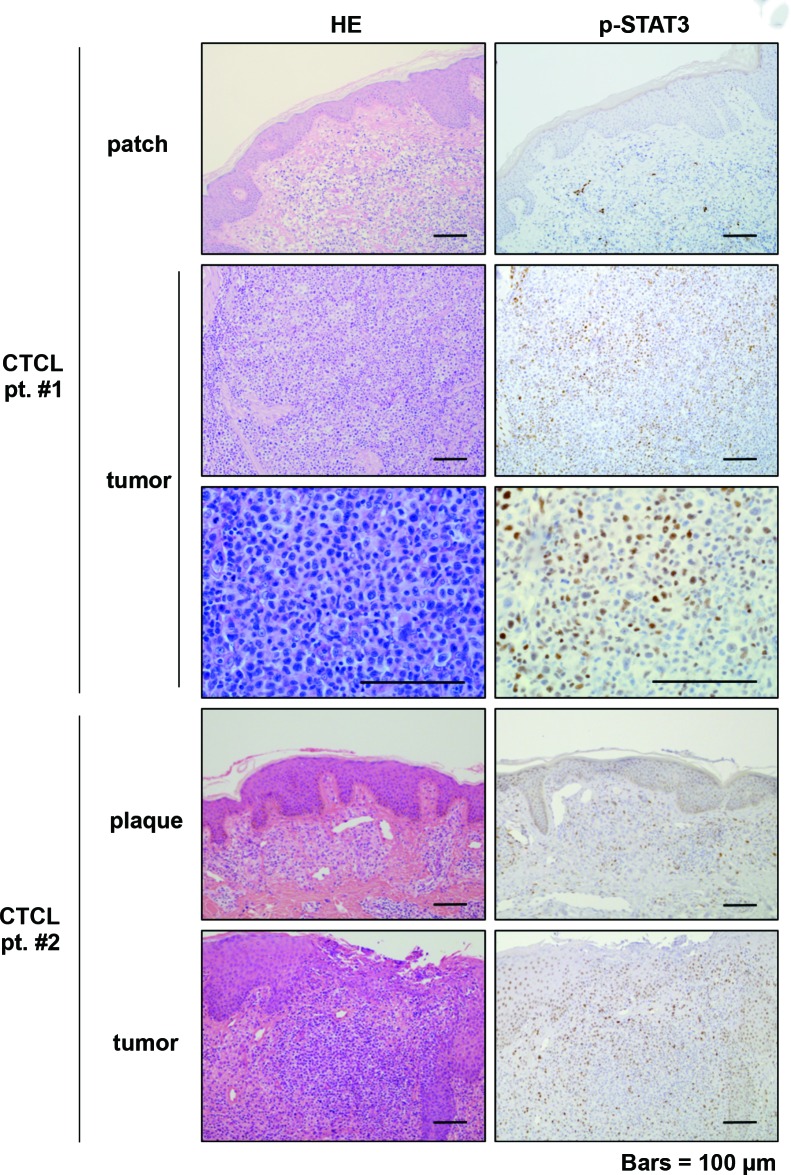
Immunohistochemical analysis of p-STAT3 in early and advanced mycosis fungoides Hematoxylin-Eosin (HE) and p-STAT3 immunohistochemical staining of primary mycosis fungoides (MF) (CTCL patient #1 and CTCL patient #2). Patch (patient #1) or plaque (patient #2) and tumor (patient #1, patient #2) of the same patients are shown. Scale bars: 100 μm.

Recently, several studies have shown that JAK-STATs (e.g., STAT3 and STAT5) play crucial roles in the pathogenesis of early to advanced stage CTCL. In particular, continuous activation of STAT3 is frequently reported in advanced CTCL [22, 23]. IL-22 is also known to induce the activation of JAK1 and Tyk2, leading to the phosphorylation of members of the STAT family [24]. Furthermore, phosphorylated STAT3 directly binds to the *CCL20* promoter, leading to a robust increase in *CCL20* transcription [25, 26]. These data suggest that the IL-22/STAT3/CCL20 signal cascade plays a crucial role in the pathogenesis of advanced CTCL. We hypothesized that in advanced CTCL, IL-22 could activate the JAK1-STAT3 pathway through activation of the IL-22 receptor (IL22RA1) and that the activation of p-STAT3 could promote *CCL20* transcription. We examined the expression of p-STAT3 and CCR6, a specific receptor of CCL20, in paraffin-embedded samples with early and advanced CTCL. p-STAT3 staining in the advanced cases was stronger than that in the early cases (Figure 1, Table 1). On the other hand, there seemed to be no difference in the expression of CCR6 between the early and advanced cases. However, accumulation of CCR6^+^ cells, which include dendritic cells and tumor cells, was more in the advanced cases than in the early cases (Supplementary Figure S1).

**Table 1:** Results of immunohistochemical analysis of p-STAT3 in 5 cases of early (patch or plaque phase) to advanced (tumor phase) CTCL

pt #	Age	Sex	CTCLsubtype	Stage	p-STAT3
1	45	M	MF*	patch	−
				tumor	+++
2	73	M	MF	plaque	+
				tumor	+++
3	67	M	MF	plaque	+
				tumor	++
4	71	M	MF	plaque	+
				tumor	+
5	62	M	MF	plaque	+
				tumor	+++

* mycosis fungoides.

### Transcriptional activation of *CCL20* by the IL-22/STAT3 cascade in CTCL cells

**Figure 2:**
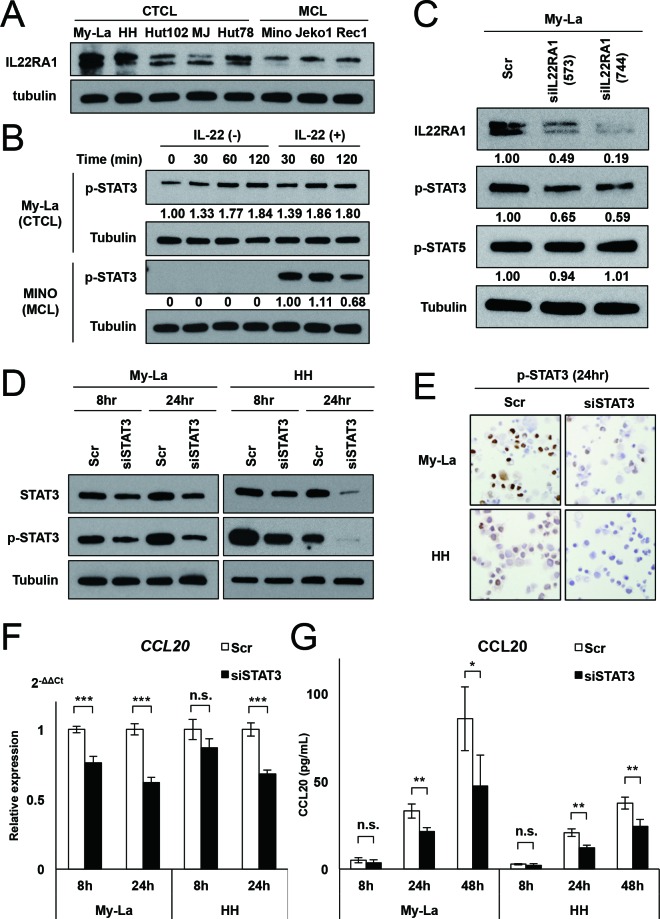
Spontaneous activation of STAT3 induces *CCL20* transcription and production **A.** Western blot analysis for IL22RA1 in the CTCL cell lines (My-La, HH, Hut102, MJ, and Hut78) and MCL cell lines (Mino, Jeko1, and Rec1). **B.** Western blot analysis for p-STAT3 in My-La and Mino cells, which were treated with IL-22 (50 ng/mL) after culture in a medium without human serum (for My-La) or bovine serum (for Mino) for 60 min. **C.** Western blot analysis for IL22RA1 in My-La cells transiently transduced with siIL22RA1 and control cells (Scrambled: Scr). **D.** Western blot analysis for STAT3 and p-STAT3 in My-La cells transiently transduced with siSTAT3 and control cells (Scrambled: Scr). **E.** Photographs (×200) of immunohistochemical staining of p-STAT3 in My-La and HH cells transduced with siSTAT3 or control (Scr). Transduced cells were stained with p-STAT3 (can be seen as brown cells in the figure). **F.** q-RT-PCR for *CCL20* in My-La and HH cells transiently transduced with siSTAT3 or control (Scr). Expression of *CCL20* in the My-La cells: 76.3% at 8 h, 62.1% at 24 h; that in the HH cells: 86.9% at 8 h, 68.2% at 24 h. Student's t test was used for examining significance. Asterisks (*) indicate statistical significance: ***P < 0.001. n.s.: not significant. Bars are means ± 95% CI (confidence interval) of three independent experiments. **G.** ELISA assay for CCL20 in My-La and HH cells transiently transduced with siSTAT3 or control (Scr). Concentration of CCL20 in the My-La cells: 70.7% at 8 h, 65.0% at 24 h, and 55.3% at 48 h; that in the HH cells: 55.1% at 8 h, 58.5% at 24 h, and 64.6% at 48 h. Student's t test was used for examining significance. Asterisks (*) indicate statistical significance: *0.01 ≤ *P* < 0.05, **0.001 ≤ *P* < 0.01, n.s.: not significant. Bars are means ± 95% CI (confidence interval) of three independent experiments.

IL-22 is known to activate STAT3 *via* interaction with its receptor complex, which consists of two different subunits (IL22RA1 and IL10RB2) in mantle cell and anaplastic large cell lymphomas [27, 28]. These reports suggest that the interaction of IL-22 with its receptor also might activate STAT3 in advanced CTCL because the IL-22 receptor was strongly expressed in advanced CTCL [21]. Firstly, we confirmed strong IL22RA1 expression in the CTCL and mantle cell lymphoma (MCL) cell lines (Figure 2A), which was consistent with our previous results and those of some other research groups [21, 27]. We next performed IL-22 treatment to examine the effect on STAT3 activation in a CTCL cell line, My-La, and a MCL cell line, Mino. This experiment was conducted using a medium without human serum (for My-La) or fetal bovine serum (for Mino). We previously demonstrated that IL-22 was spontaneously increased in culture medium of My-La cells in a time-dependent manner [21]. Although we could not find any change in the expression of p-STAT3 after IL-22 stimulation, the level of p-STAT3 also spontaneously increased in a time-dependent manner in My-La cells. On the other hand, we found that p-STAT3 activation in the Mino cells was dependent on IL-22 stimulation (Figure 2B). This result suggests that activation mechanism of STAT3 might be different by distinct lymphoma subtypes. Furthermore, we found that transient knockdown of IL22RA1 downregulated the expression of p-STAT3 but not p-STAT5 in My-La (Figure 2C), demonstrating the presence of IL22RA1 is required for the phosphorylation of STAT3 in the cell line.

To examine whether knockdown of STAT3 might decrease the expression and production of CCL20, we performed the transient knockdown of STAT3 (siSTAT3) in the CTCL cells (Figure 2D and 2E). The results of q-PCR indicated that siSTAT3 downregulated *CCL20* expression (Figure 2F). In agreement with the results of q-PCR, the results of the ELISA assay also showed that siSTAT3 inhibited CCL20 production (Figure 2G). In addition, we could not detect any significant difference in the cell counts between the scramble control and siSTAT3 during a 24-hour observation in My-La and HH cells (not shown). Together, these results indicate that the spontaneous activation of IL22RA1-STAT3 occurs in advanced CTCL, and that it induces CCL20 production. This may be due to occurrences of genetic mutation of gene(s) of JAK/STAT3 cascade, although detection for mutation of this cascade might be needed for the further study.

### *In vivo* metastasis-inhibitory effects of siCCL20 or siCCR6 in CTCL mice

**Figure 3:**
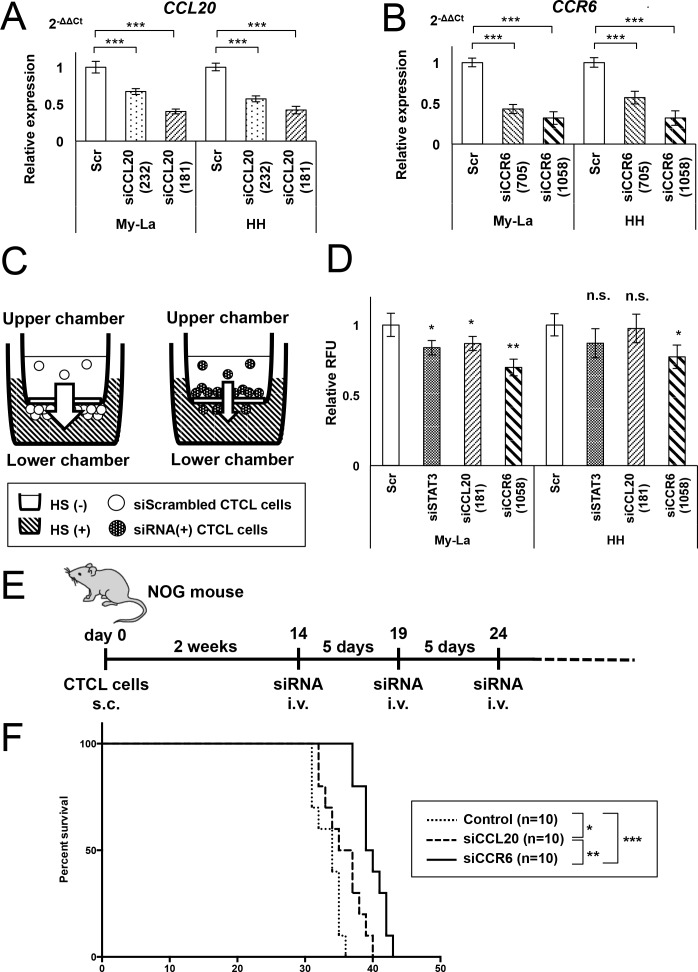
Administration of siCCL20 or siCCR6 significantly inhibits CTCL cell metastasis *in vivo* **A.** q-RT-PCR for *CCL20* in My-La or HH cells transiently transduced with siCCL20 (181), siCCL20 (232), and control siRNA (Scr) for 24 h. The expression observed was as follows: 40.0% and 42.1% in siCCL20 (181)-transduced My-La and HH cells, respectively; 66.7% and 57.4% in siCCL20 (232)-transduced My-La or HH cells, respectively. **B.** q-RT-PCR for *CCR6* in My-La or HH cells transiently transduced with siCCR6 (1058), siCCR6 (705), and control siRNA (Scr) for 24 h. The expression observed was as follows: 32.4% and 32.0% in siCCR6 (1058)-transduced My-La and HH cells, respectively; 43.0% and 57.1% in siCCR6 (705)-transduced My-La and HH cells, respectively. **C.** Schematic illustration of the migration assay using siRNAs. HS: human serum. **D.** Migration assay of My-La and HH cells transiently transduced with siSTAT3, siCCL20, siCCR6, and control (Scr). Changes in the migration of the cells were determined relative to that observed with RFU (480 nm/520 nm, relative fluorescent unit); the RFU of cells treated with scrambled (control) was standardized as 1.0. Student's t test was used for examining significance. Asterisks (*) indicate statistical significance: *0.01 ≤ *P* < 0.05, **0.001 ≤ *P* < 0.01, n.s.: not significant. Bars are means ± 95% CI (confidence interval) of three independent experiments. **E.** Schematic illustration of the *in vivo* protocol of siCCL20 or siCCR6 injection in NOG mice inoculated with My-La cells. **F.** Kaplan-Meier survival curve of the mouse model of human CTCL inoculated with siCCL20 or siCCR6 are also shown. Log-rank test was used for examining significance. Asterisks (*) indicate statistical significance: *0.01 ≤ *P* < 0.05, **0.001 ≤ *P* < 0.01, ***P < 0.001.

Based on the observations as shown in Figures 1 and 2, the STAT3-CCL20 cascade is crucially involved in the pathogenesis of CTCL. We previously showed that interaction of CCL20 and CCR6 is also important for the metastasis and invasion of CTCL cells [21]. Thus, the inhibition of CCL20-CCR6 interaction could be a new therapeutic strategy against metastatic CTCL. To test this hypothesis, we tried to inhibit CCL20-CCR6 interaction using siCCL20 and siCCR6. The knockdown of CCL20 or CCR6 resulted in a significant reduction in the expression of *CCL20* or *CCR6* in the siRNA-treated cells, respectively (Figure 3A and 3B). We then carried out *in vitro* migration assays using My-La and HH transfectants plated on transwell plates (Figure 3C). In this experiment, the migration from the upper chamber to the lower chamber of the transwell plate was triggered by 2% human serum in the lower chamber. Using the transwell assay system, we have previously shown that the chemotaxis of CTCL cells is nutrition dependent [21]. Therefore, after incubating the cells for 16 h, we measured the migration index (RFUs) to the lower chamber in our migration assay performed using siSTAT3, siCCL20, and siCCR6. All three siRNAs were effective in reducing cell migration, with siCCR6 being the most effective one (Figure 3D). These effects were also visually confirmed as shown in Supplementary Figure S2A and B.

Therefore, we conducted *in vivo* administration of these siRNAs including siCCR6 or siCCL20 in our established mouse model, named the CTCL mouse. We had previously showed that the inoculation of the My-La cell line in NOG mice resulted in death due to metastasis in multiple visceral organs [21]. In this study, we injected siCCL20 or siCCR6 with atelocollagen from through the tail vein every 5 days following day 14 after My-La injection (Figure 3E), and found that the administration of either siCCL20 or siCCR6 in the CTCL mice resulted in significantly prolonged survival than that observed in the controls (Figure 3F). These results suggest that the quantitative reduction in CCL20 and CCR6 levels due to administration of the respective siRNAs might contribute to the prolonged survival in the CTCL mice.

### *In vivo* metastasis-inhibitory effect of the neutralizing CCL20 antibody in CTCL mice

**Figure 4:**
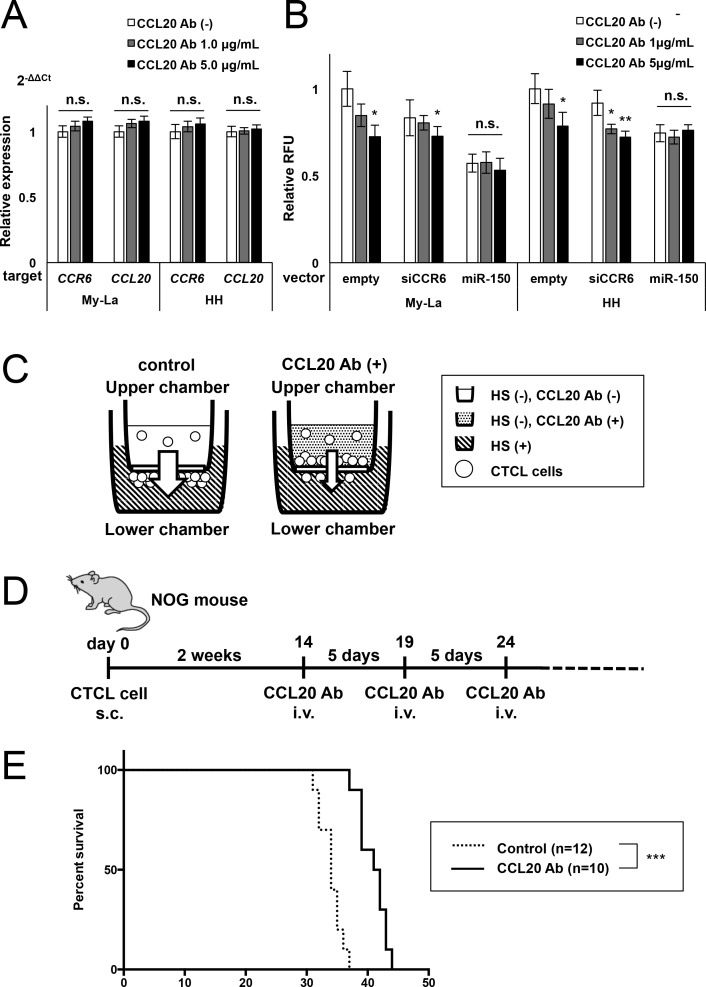
Migration inhibition by neutralizing CCL20 antibody in CTCL cells **A.** q-RT-PCR for *CCR6* and *CCL20* in My-La and HH cell lines treated with neutralizing CCL20 antibody (described “CCL20 Ab” in this Figure) for 24 h. n.s.: not significant. **B.** Migration of the CTCL cells treated with neutralizing CCL20 antibody against My-La or HH cells stably transduced with siCCR6 or miR-150 or control (GFP-empty). The RFU of cells treated with GFP-empty was standardized as 1.0. Student's t test was used for examining significance. Asterisks (*) indicate statistical significance: *0.01 ≤ *P* < 0.05, **0.001 ≤ *P* < 0.01. n.s.: not significant. Bars are means ± 95% CI (confidence interval) of three independent experiments. **C.** Schematic illustration of the migration assay using neutralizing CCL20 antibody is shown. HS: human serum. **D.** Schematic illustration of the *in vivo* protocol of neutralizing CCL20 antibody injection in NOG mice inoculated with My-La cells. **E.** Kaplan-Meier survival curve of the mouse model of human CTCL metastasis. NOG mice were inoculated with neutralizing CCL20 antibody. Log-rank test was used for examining significance. Asterisks (*) indicate statistical significance: ***P < 0.001.

The prolonged survival of the NOG mice injected with siCCL20 or siCCR6 might reflect the inhibition of CCL20-CCR6 interaction by the quantitative reduction in the expression of CCL20 and CCR6. We further examined whether neutralizing antibody against CCL20 (neutralizing CCL20 antibody) could interfere with the reciprocal interaction between CCL20 and CCR6 in CTCL mice. We also performed *in vitro* examination using the neutralizing CCL20 antibody to elucidate whether neutralization of CCL20 could block its interaction against CCR6 in CTCL cells. The expression of *CCL20* and *CCR6* in CTCL cells treated with neutralizing CCL20 antibody did not find significant differences between the control and treated cells (Figure 4A). Next, we conducted a migration assay with the upper chamber including CTCL cells (My-La or HH) with neutralizing CCL20 antibody in the medium. The cells found to be GFP-empty, and were stably transduced with siCCR6 or miR-150. The GFP-empty and siCCR6-transduced CTCL cells showed significant reduction in migration toward human serum in the lower chamber after treatment with neutralizing CCL20 antibody in a dose-dependent manner. However, the miR-150-transduced cells showed a significant reduction in CCL20 and CCR6 production when compared with a single knockdown of either CCL20 or CCR6 [21]. Further, treatment with neutralizing CCL20 antibody also did not seem to inhibit the migration of these cells (Figure 4B and 4C). *In vivo* administration of neutralizing CCL20 antibody in CTCL mice resulted in prolonged survival of the xenografted mice (Figure 4D and 4E). These results indicate that the neutralizing CCL20 antibody also contributed to the survival of the CTCL mice by interfering with CCL20-CCR6 interaction without quantitative reduction of CCL20 and CCR6 expression.

**Figure 5:**
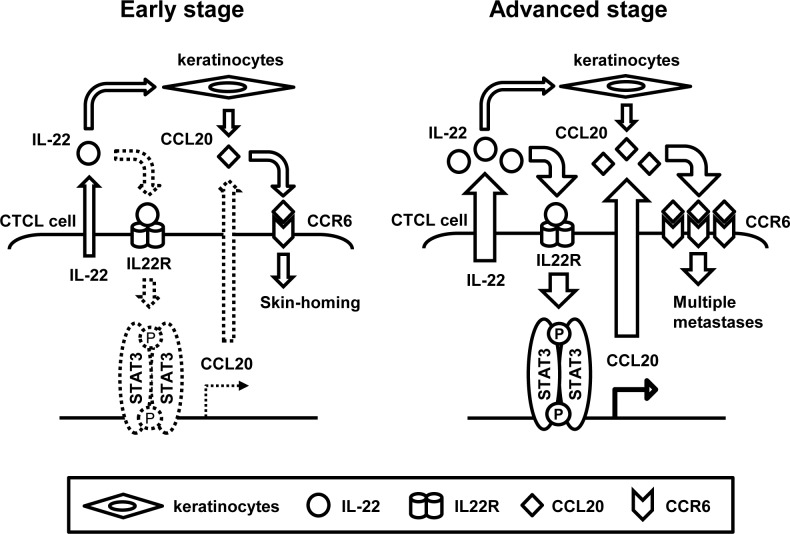
Schematic illustrations of IL-22/STAT3/CCL20 signaling in early and advanced CTCL

Our present data lead us to conclude the following idea: in early CTCL, the production of CCL20 in the tumor cells is low; therefore, keratinocytes are a major source of CCL20. Thus, early CTCL cells might home to the skin. However, in accordance with the increased STAT3 activation observed during disease progression, the production of CCL20 by tumor cells also increases and serum CCL20 levels are elevated [20]. Finally, in advanced cases, CTCL cells may expand themselves without organ selectivity (Figure 5).

In this study, we took the first steps towards exploring whether neutralizing CCL20 antibody might have therapeutic potential against CTCL by evaluating its effects on mice xenografted with CTCL cells *in vivo*. It has previously been reported that CCL20-CCR6 interaction could be a therapeutic target for metastatic solid tumors [29-31]. In addition, the possibility of using this interaction as a therapeutic target in autoimmune diseases such as rheumatoid arthritis has also been suggested [32, 33]. In CTCL lesional skins, keratinocytes produce CCL20 and induce the chemotaxis of various immune competent cells such as dendritic cells that express CCR6, which could lead to inflammation of the surrounding tissue [20]. Moreover, CCL20 levels in advanced CTCL are usually high [20]. Thus, an additional effect of neutralizing CCL20 antibody is also suggested: it might not only inhibit CCL20-CCR6 interaction in metastatic CTCL cells but might also decrease the activation of this interaction in surrounding cells, which is considered important for tumor establishment. Our findings strongly suggest that inhibition of CCL20-CCR6 interaction could be crucial therapeutic strategy for advanced CTCL.

## MATERIALS AND METHODS

### Primary samples

Immunohistochemistry of p-STAT3 and CCR6 was performed for 5 patients with MF. Two different types of skin lesions from the same patients were used. Detailed information was described in Table 1. Samples were collected under a protocol approved by the Institutional Review Boards of Akita University and University of Tokyo.

### Cell lines

My-La was purchased from the European Collection of Cell Culture (ECACC). HH, Hut78, Hut102, MJ, Mino, Jeko1, and Rec1 were purchased from the American Type Tissue Collection (ATCC). CTCL cell lines (My-La, HH, Hut78, Hut102, and MJ) were cultured in Artemis-1 medium (with or without 2% inactivated human serum), which is a chemically defined, serum-free medium purchased from NihonTechno Service Co. Ltd. (Ibaraki, Japan). It contains recombinant human insulin (5 μg/L), recombinant human IL-2 (250 IU/mL), human serum albumin (2 g/L), and no other cytokine nor growth factor are contained. MCL cell lines (Mino, Jeko1, and Rec1) were cultured with RPMI1640 plus 10% FCS. We have confirmed the identity of all cell lines recently (April 2015) by short tandem repeat analysis (Takara Bio CDM Center).

### *In vivo* administration of siRNA or antibody against CTCL mice

My-La cells (2×10^5^/per body) were subcutaneously injected into the right or left side of the body of 6- to 8-week-old female NOD/Shi-scid IL-2γnul (NOG) mice (Central Institute for Experimental Animals, Kawasaki, Japan) [34]. All of injected mice could be dead after 31-37 after injection [21]. 200μL of siRNA or scrambled (control) plus atelocollagen (30μM) [35] were injected into tail vain of NOG mice after day 14 from CTCL cells transplantation. Injection was conducted with every 5 days. Neutralizing CCL20 antibody (5mg/kg, MAB360, R&D systems, Minneapolis, MN, USA) or isotype-IgG control were also injected against transplanted NOG mice with every 5 days after day 14 from CTCL cells transplantation.

### Western blot analysis

Antibodies of STAT3 (#4904), p-STAT3 (#9145), and p-STAT5 (#4322) were all purchased from Cell Signaling Technology (Danvers, MA, USA). IL22RA1 (ab5984) and CCR6 (ab137369) were purchased from Abcam (Cambridge, UK). Tubulin (MS-581-P0) was from NeoMarkers (Fremont, CA, USA).

### Transient siRNA transfection

For transient knockdown of *IL22RA1*, *CCR6*, and *CCL20*, we used respective two siRNA-double strands targeting different sites on their mRNA. These two double strand pairs of siRNAs and negative control siRNA (scrambled siRNA) were designed and synthesized by Nippon Gene (Toyama, Japan). Detailed sequence information is described in Supplementary Table S1. For transient knockdown of *STAT3*, we used Dharmacon ON-TARGET plus SMART pool STAT3 siRNA (#L-003544-00). For non-targeting control, we used Dharmacon ON-TARGETplus Non-targeting Pool (#D-001810-10). SMART pool siRNA contains a pool of four siRNA sequences against the target gene. These were purchased from GE Healthcare Dharmacon (Lafayette, CO, USA). The transfection of siRNA was used by the Nucleofector II and the Cell Line Nucleofector Kit V (VCA-1003) (Amaxa, Koeln, Germany) according to the manufacturer's protocol. Briefly, the cells were resuspended in the nucleofector V solution. 100μL of cell suspension at a density of 1×10^7^/mL mixed with a total of 1.0μg of siRNA were transferred to a cuvette and nucleofected with an Amaxa Nucleofector II apparatus using “A-023” program.

### Stable siRNA or microRNA transfection

As for stable miR-150 or siCCR6 transfectants, please see our previous paper [21].

### Real time quantitative PCR analysis

Real time quantitative PCR (q-RT-PCR) was performed by use of Taqman method (Applied Biosystems, Foster City, CA, USA). Taqman probes of *CCL20, CCR6*, and *GAPDH* were purchased from Applied Biosystems. Gene expression levels were separately normalized with *GAPDH*, and the relative expression level of specific mRNA was presented by 2^−ΔΔCt^. The −ΔΔC_t_ value was then calculated by subtracting the −ΔC_t_ value for a each control sample from the respective −ΔC_t_ values from the objected cells. Quantitative RT-PCR was performed using Light Cycler Nano (Roche) with the Taqman method. Total RNA was extracted using TRIzol (Life technologies, Palo Alto, CA, USA). Reverse transcription was performed using a Transcriptor First Strand cDNA Synthesis Kit (Roche).

### Enzyme-linked immunosorbent assay (ELISA)

Human CCL20/MIP-3α Quantikine ELISA Kit (DM3A00, R&D system, Inc. Minneapolis, MN) was used for ELISA by manufactualer's protocol.

### Cell migration assay

*In vitro* cell migration assay was carried out by use of CytoSelect 96-Well Cell Migration Assay kit (5μm, Fluorometric Format) (Cell Biolabs, Inc. SanDiego, CA, USA) according to the manufacturer's protocol. Briefly, 1×10^6^ cells in 100μL of medium without human serum in the upper chamber through a coated basement membrane toward to 2% human serum at lower chamber (150μL). Incubate time was 16 hours and cell lysis buffer were transferred to 96 well plate and fluorescence was measured by plate reader at 480 nm/520 nm (RFU: relative fluorescent unit).

### Study approval

The protocols for animal experimentation described in this paper were previously approved by the Animal Committee of Akita University (accept number a-1-2301). The “Regulations for Animal Experimentation” of the university were completely adhered to in all animal experiments. Written informed consent was obtained from all patients prior to collection of specimens, in keeping with all institutional policies and according to the Declaration of Helsinki. Samples were collected under a protocol approved by the Institutional Review Boards of Akita University (no.1313) and University of Tokyo (no.10746).

### Statistical analysis

Log-lank test method and Student's t test were used for examining significance. In this presentation, asterisks (*) indicate statistical significance: *0.01 ≤ *P*<0.05, **0.001≤ *P*<0.01, ***P<0.001.

## SUPPLEMENTARY MATERIAL TABLE AND FIGURES


