# Supplementary material for: Disruption of CCL20-CCR6 interaction inhibits metastasis of advanced cutaneous T-cell lymphoma
Source: Oncotarget. 2016 Jan 14;7(12):13563–74. doi: 10.18632/oncotarget.6916 (PMC4924661; doi:10.18632/oncotarget.6916)
Supplement: Supplementary file 1 [file oncotarget-07-13563-s001.pdf]

Disruption of CCL20-CCR6 interaction inhibits metastasis of advanced cutaneous T-cell lymphoma

Supplementary Material

Supplemental Table S1. Sequence information of siIL22RA1, siCCL20, and siCCR6.

siRNA	probe	5'-3'
siIL22RA1(537)	sense	CCUGAUGUGACCUGUAUCUdTdT
	antisense	AGAUACAGGUCACAUCAGGdTdT
siIL22RA1(744)	sense	GGGAAGCAGAGAGAAUAUGdTdT
	antisense	CAUAUUCUCUCUGCUUCCCdTdT
siCCR6(705)	sense	CUGCUCCUGACUUGCAUUAdTdT
	antisense	UAAUGCAAGUCAGGAGCAGdTdT
siCCR6(1058)	sense	AGCCAUCCGUGUAAUCAUAdTdT
	antisense	UAUGAUUACACGGAUGGCUdTdT
siCCL20(181)	sense	CCGUAUUCUUCAUCCUAAAdTdT
	antisense	UUUAGGAUGAAGAAUACGGdTdT
siCCL20(232)	sense	UGAAGGCUGUGACAUCAAUdTdT
	antisense	AUUGAUGUCACAGCCUUCAdTdT

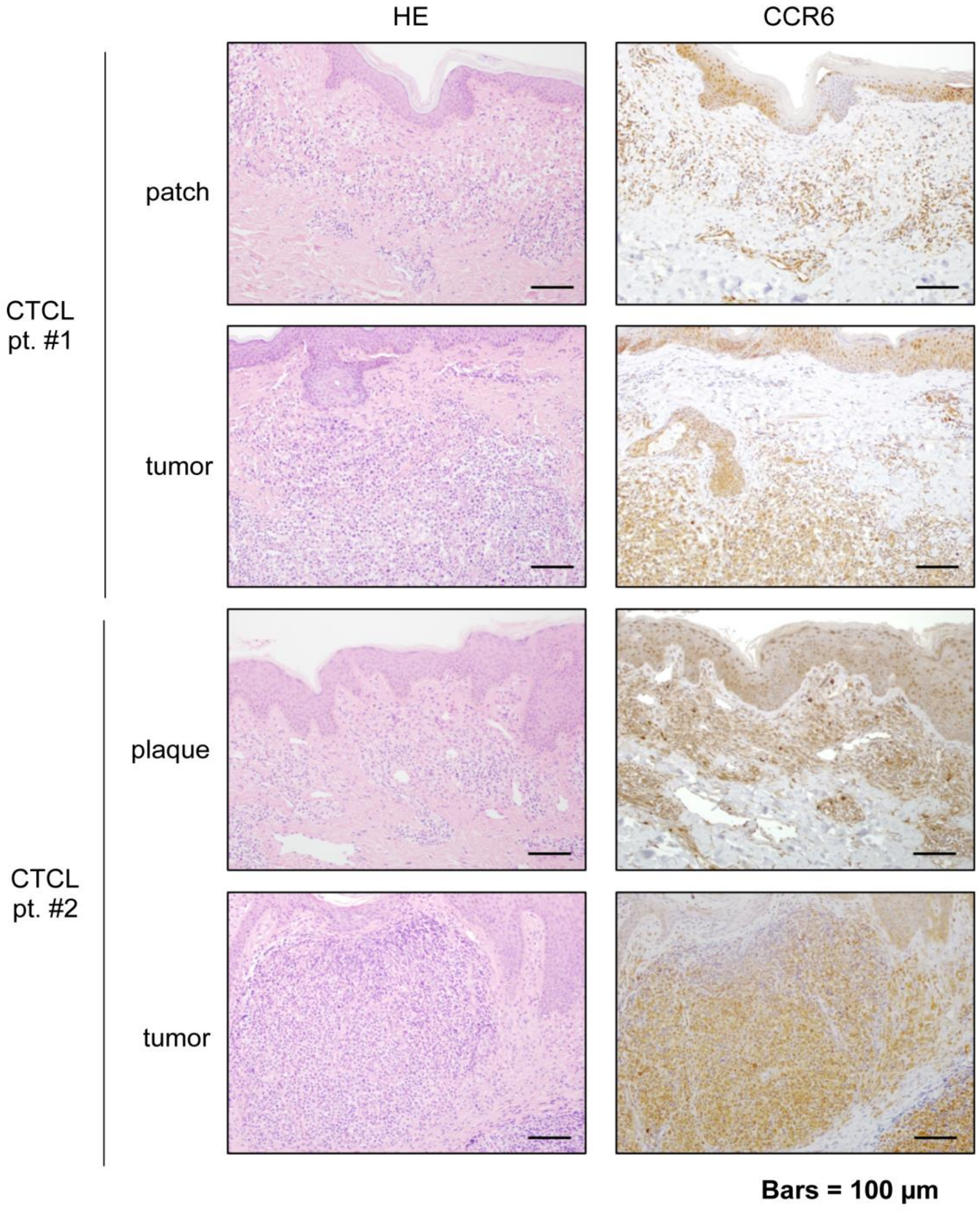
HE
CCR6
patch
tumor
CTCL
pt. #1
plaque
tumor
CTCL
pt. #2
Bars = 100 μm

Supp Fig1:

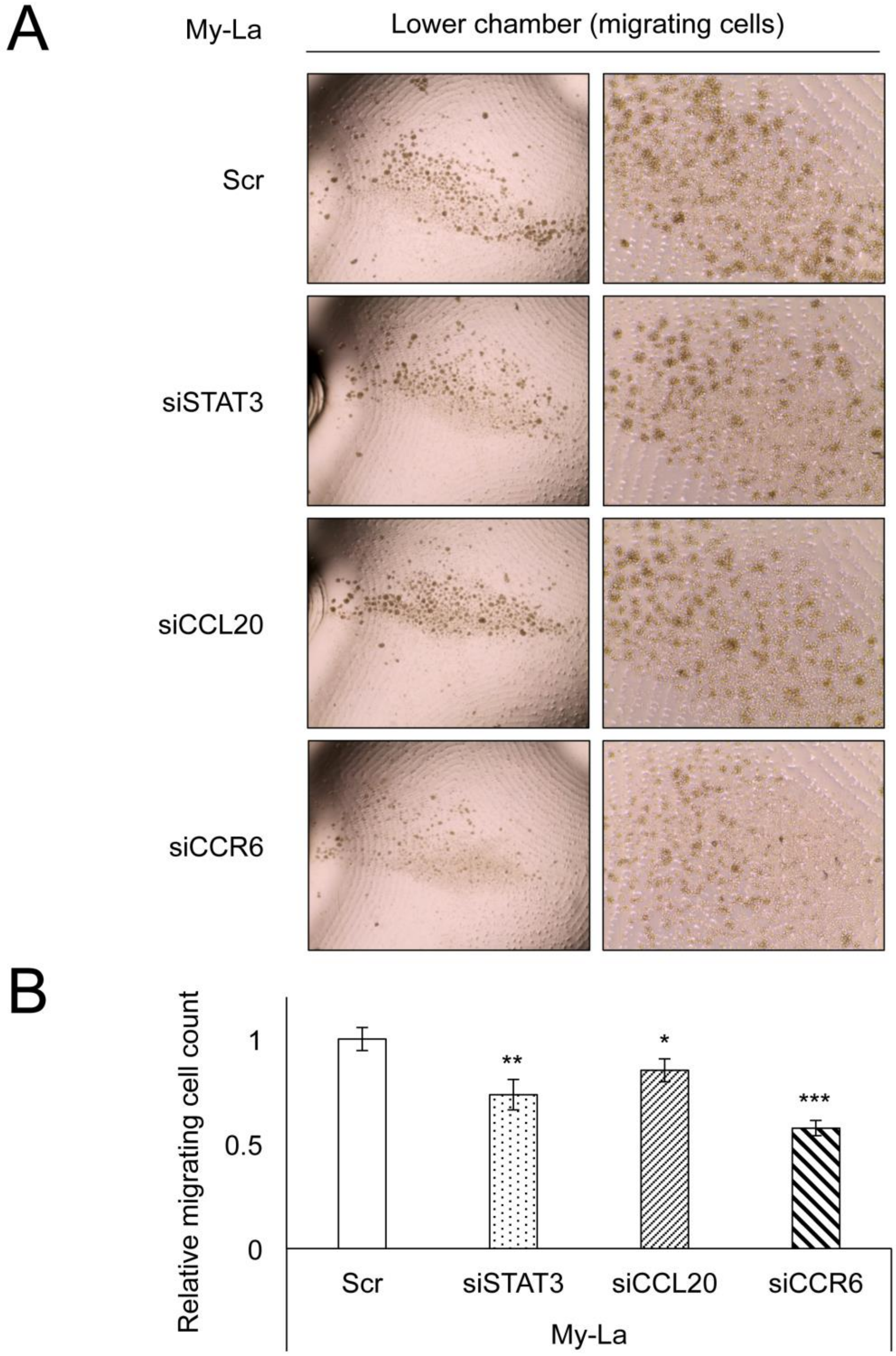
A
My-La
Lower chamber (migrating cells)
Scr
siSTAT3
siCCL20
siCCR6
B
Relative migrating cell count
1
0.5
0
**
*
***
Scr
siSTAT3
siCCL20
siCCR6
My-La

Supp. Fig. 2:
